# Mindcraft, a Mobile Mental Health Monitoring Platform for Children and Young People: Development and Acceptability Pilot Study

**DOI:** 10.2196/44877

**Published:** 2023-06-26

**Authors:** Balasundaram Kadirvelu, Teresa Bellido Bel, Xiaofei Wu, Victoria Burmester, Shayma Ananth, Bianca Cabral C C Branco, Braulio Girela-Serrano, Julia Gledhill, Martina Di Simplicio, Dasha Nicholls, A Aldo Faisal

**Affiliations:** 1 Brain & Behaviour Lab Department of Computing and Department of Bioengineering Imperial College London London United Kingdom; 2 Division of Psychiatry, Department of Brain Sciences Imperial College London London United Kingdom; 3 Chair in Digital Health Faculty of Life Sciences University of Bayreuth Bayreuth Germany

**Keywords:** mobile mental health, passive sensing, smartphone apps for mental health, children and young people, adolescents, digital tools, mobile apps

## Abstract

**Background:**

Children and young people's mental health is a growing public health concern, which is further exacerbated by the COVID-19 pandemic. Mobile health apps, particularly those using passive smartphone sensor data, present an opportunity to address this issue and support mental well-being.

**Objective:**

This study aimed to develop and evaluate a mobile mental health platform for children and young people, Mindcraft, which integrates passive sensor data monitoring with active self-reported updates through an engaging user interface to monitor their well-being.

**Methods:**

A user-centered design approach was used to develop Mindcraft, incorporating feedback from potential users. User acceptance testing was conducted with a group of 8 young people aged 15-17 years, followed by a pilot test with 39 secondary school students aged 14-18 years, which was conducted for a 2-week period.

**Results:**

Mindcraft showed encouraging user engagement and retention. Users reported that they found the app to be a friendly tool helping them to increase their emotional awareness and gain a better understanding of themselves. Over 90% of users (36/39, 92.5%) answered all active data questions on the days they used the app. Passive data collection facilitated the gathering of a broader range of well-being metrics over time, with minimal user intervention.

**Conclusions:**

The Mindcraft app has shown promising results in monitoring mental health symptoms and promoting user engagement among children and young people during its development and initial testing. The app's user-centered design, the focus on privacy and transparency, and a combination of active and passive data collection strategies have all contributed to its efficacy and receptiveness among the target demographic. By continuing to refine and expand the app, the Mindcraft platform has the potential to contribute meaningfully to the field of mental health care for young people.

## Introduction

Mental health issues among children and young people (CYP) have emerged as a significant public health concern [1]. A 2021 NHS digital survey in the United Kingdom revealed that 17.4% of CYP aged 6-16 years experienced at least 1 mental disorder, with a rising trend in emotional disorders such as depression and anxiety since 2017 [2]. The COVID-19 pandemic and its associated social isolation, public health disruptions, and economic decline are expected to exacerbate these issues, as seen in previous large-scale disasters [3]. Peer-to-peer contact is critical for social species [4], especially for young people who may be more affected by social distancing due to COVID-19. Research shows that isolation and deprivation of social interaction during adolescence can cause behaviors resembling depression, anxiety, and even impairment of certain cognitive skills [5-7]. A mental illness epidemic is predicted to emerge following the extended period of COVID-19–related behavioral changes [8], making prompt and comprehensive mitigation planning essential to address long-term mental health consequences.

Smartphone usage is widespread among CYP, with 88% of 13- to 18-year-olds in the United States owning smartphones [9]. This ubiquity makes smartphone apps a promising option for supporting CYP's mental health [10,11]. To reduce the widening mental health treatment gap and supplement in-person treatments, further research and investment in mobile health apps are increasingly necessary.

In recent years, the mental health field has experienced a surge in applications designed for monitoring mental health [12]. A limitation of many of these applications is their reliance on self-reported data, often referred to as active data, which may be subjected to personal biases and potentially lead to misleading conclusions in decision-making [13,14]. To address this issue, researchers have explored the usage of mobile phone sensor data, such as GPS, accelerometer, microphone, and battery usage. These data can be collected passively, without active user participation (hence referred to as passive data) and with minimal user burden.

Smartphones' passive sensing capabilities facilitate understanding of user behavior and automatic personality detection, eliminating the need for investigating hard-to-measure characteristics. Studies have showed that passive data can objectively reflect users' emotional state and generate profiles of temperament and behavior [15,16]. One study used various smartphone data from 83 individuals over 8 months to predict Big Five personality traits with 69%-76% accuracy [17]. Another study used passive data to classify 166 users from 5 countries into Big Five traits with 63%-71% accuracy, observing increased accuracy in gender- and country-specific models [18]. The potential for passive data to predict personality traits renders smartphone apps a promising avenue for influencing user behavior and providing personalized mental health support.

Most studies investigating passive data use in apps have focused on adult populations, with limited research on youth [19,20]. Furthermore, given the rising concerns about data privacy, especially among young adults [21], understanding how adolescents engage with mental health apps, particularly how comfortable would this population be in terms of passive data sharing to monitor and improve their mental health, is essential. Consequently, we have developed Mindcraft, a mobile mental health platform for CYP, which integrates passive sensor data monitoring with self-reported updates through an engaging user interface (UI). By developing such a tool, we aim to enhance mental health monitoring and empower young adults to proactively manage their mental well-being.

This paper details the design and development of Mindcraft, along with the results obtained from a pilot study assessing the app's usability and functionality. These findings will contribute to the refinement of the platform and inform future research in the realm of mental health applications for young adults.

## Methods

### The Mindcraft App

#### Overview

**Figure 1:**
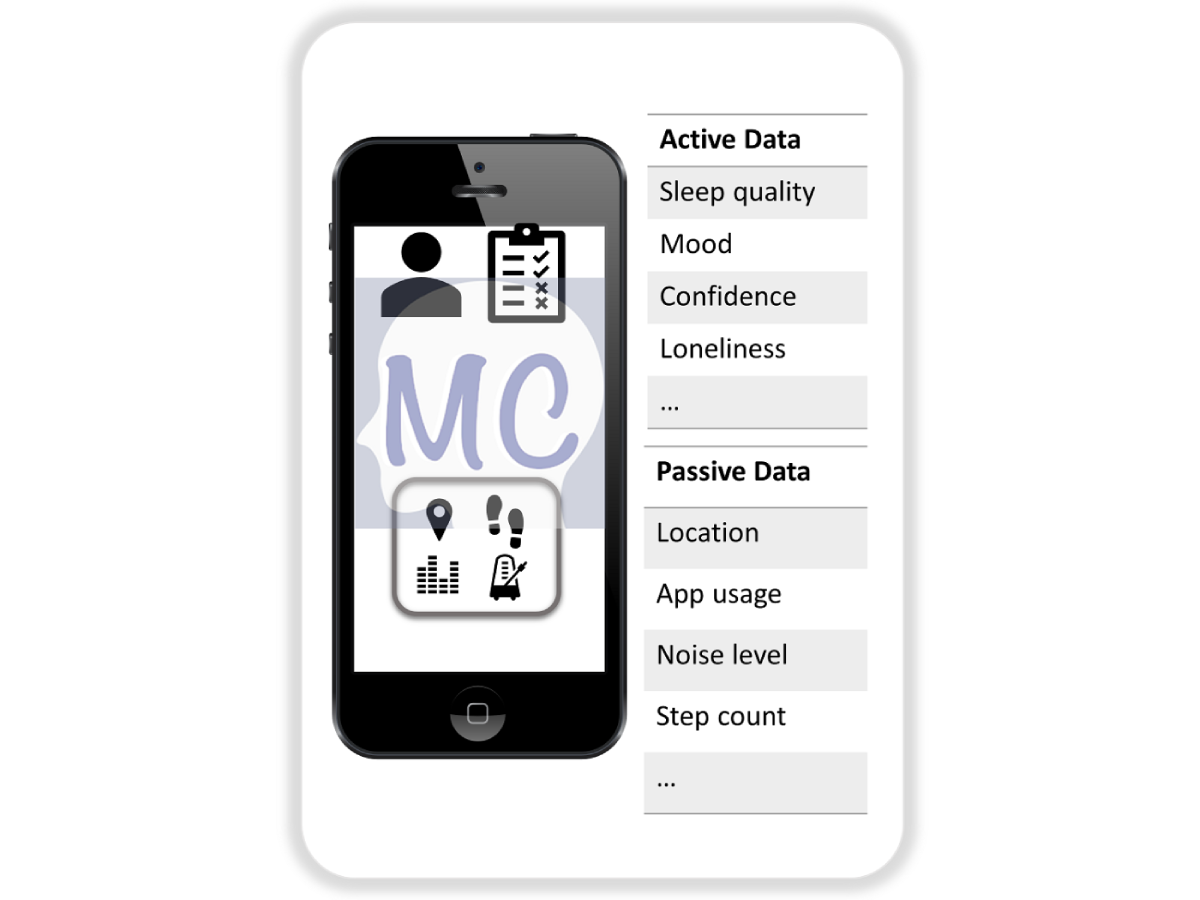
Overview of Mindcraft, a mobile mental health monitoring platform that combines self-reported questionnaire data with passive mobile sensors to monitor mental well-being markers of children and young people.

The Mindcraft app (Figure 1) is a user-friendly mobile app designed specifically for CYP to effectively monitor their mental well-being. The app provides a modern and appealing user experience to encourage participation and is designed with a modular and adaptable framework to facilitate future improvements and support various study questions. The primary objectives of the Mindcraft app are (1) combining self-reported well-being updates (active data) with phone sensor data (passive data) for effective monitoring of mental health–related behavior, (2) providing an easy-to-use and intuitive UI that promotes user participation and facilitates data collection, (3) ensuring user data are handled according to good clinical practice standards [22] for privacy and security, and (4) continuously developing and updating the app based on participant feedback and usage patterns.

The Mindcraft app features a modular design, enhancing user engagement by providing customization options to meet individual needs and interests. This design contributes to improved user experience and overall app effectiveness. The key modular components include:

1. Customizable active questions: Mindcraft targets a general population, so users have different well-being goals. The app allows users to select daily active questions tailored to their unique mental health objectives.
2. Configurable passive data: Users can choose which passive data they wish to share, ensuring that they have control over their privacy.

**Figure 2:**
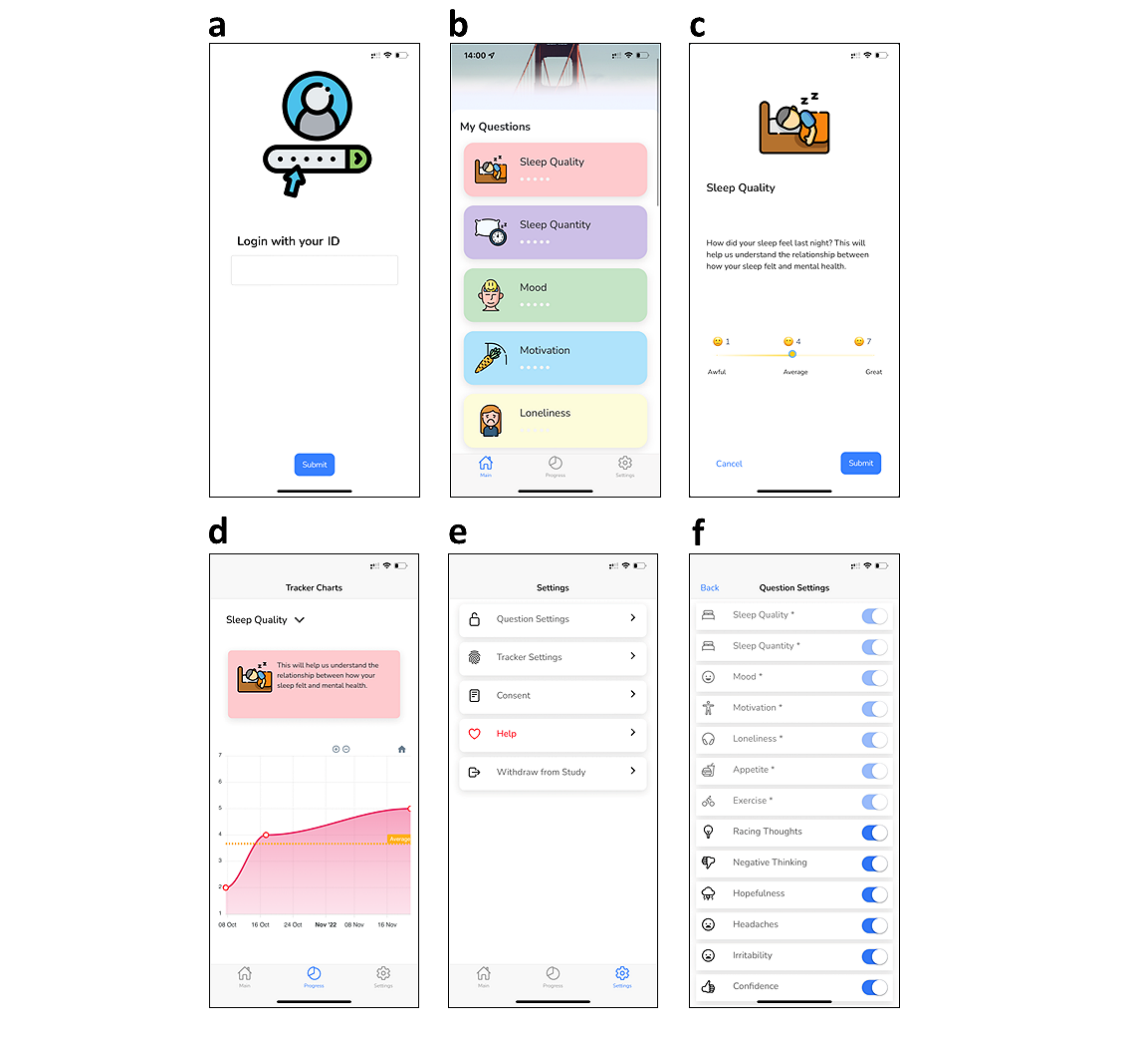
User interface of the Mindcraft app. (A) Login tab, (B) main tab, (C) example of an active questionnaire involving slider-based data entry, (D) progress tab, (E) settings tab, and (F) optional data collection settings allowing users to control what information is collected (actively and passively).

Users can set their active and passive data sharing preferences during the initial onboarding process and also can modify them at any time through the app's settings. Mindcraft app is available for free download from the App Store [23] and Google Play Store [24]. A valid study ID code is required to use the app. The Mindcraft app comprises 3 main tabs in its UI (Figure 2A-E): the main tab, the progress tab, and the settings tab, which are described below.

#### Main Tab

Mindcraft is easy to use and features an attractive design that captures users' attention. The app incorporates visual representations of users' mood and mental well-being markers, which have been shown to increase engagement [25,26]. The main tab, illustrated in Figure 2B, is the first view of the app when launched by a registered user. Here, the user can self-report a questionnaire related to their mood and well-being. The questionnaire contains 7 compulsory questions (sleep quality, sleep quantity, mood, motivation, loneliness, appetite, and exercise), as well as 11 optional questions (racing thoughts, negative thinking, hopefulness, headaches, irritability, confidence, sociability, energy levels, productivity, self-care, leisure, and a custom metric called “your measure”). The user can manage the optional questions through the settings tab. Each question can only be answered once a day; thus, the question will disappear after each submission. Most of the self-reported questions (such as mood and sleep quality) are on a scale of 1-7 and contain a slider. An example of such a question is shown in Figure 2C. Other questions (such as sleep quantity and exercise time) collect numerical data from textboxes, and the input is type-checked to ensure only valid inputs are stored (eg, numerical numbers and not text).

#### Progress Tab

The progress tab (shown in Figure 2D) charts the self-reported updates given by the user throughout the study. Selecting the data of interest causes the graph view to display the historical data and the average score for the selected period.

#### Settings Tab

In the settings tab (illustrated in Figure 2E), the user can manage the active and passive data settings, read the privacy policy, as well as withdraw from the study. As shown in Figure 2F, the app allows the user to enable or disable the optional active data questions most relevant to them. Although the focus of the app is helping the users to develop better habits of self-care, the app must not delay seeking for professional help. The help option under the settings tab provides the ability for the user to seek support when needed. To facilitate this, Mindcraft lists 4 external organizations—Shout [27], YoungMinds [28], Best For You NHS [29], and Kooth [30]—along with a brief description of each organization and links to their websites.

### System Architecture

#### Overview

**Figure 3:**
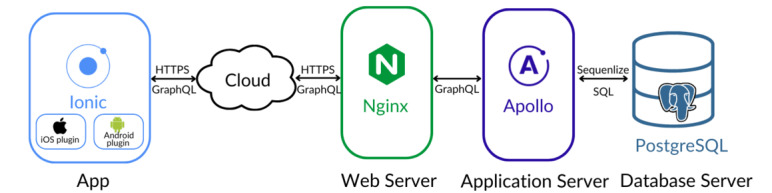
Overview of the system architecture of the Mindcraft platform.

The system architecture of Mindcraft is presented in Figure 3.

#### App

Mindcraft is a cross-platform smartphone app with a single codebase that runs on iOS and Android. The app was developed using the ionic framework as a hybrid app. Functionalities common to both mobile operating systems (user login, active data collection and transmission, progress display, and settings) were written with web technologies (TypeScript, HTML, and cascading style sheets). Mobile operating system–specific functionalities (passive data collection and local notifications) were developed, using native plugins that used Capacitor written in Swift for iOS and Java for Android. Most passive data are collected every 15 minutes, and Table 1 lists the passive data collected on each mobile operating system.

**Table 1:** Passive sensing data collected by the Mindcraft app.

Passive sensor data	Mobile operating system supported
Location^a^	iOS and Android
Step count	iOS and Android
Background noise levels^b^	iOS and Android
Ambient light	iOS and Android
Battery level	iOS and Android
App usage on the phone	Android only

^a^iOS: collected when there is a significant change in the user's location.

^b^iOS: collected only when the app is in the foreground.

#### Server

The backend server has a 3-layer architecture: web server layer, application layer, and database layer (Figure 3). We used Nginx as the web server to support reverse proxy and load balancing to increase the security and scalability of the app. For the application layer, we used Apollo Server, an open-source GraphQL server, to process the entire backend application logic. PostgreSQL is used as the database layer to store user information, active data, and passive data. The 3 layers are also containerized using separate docker containers to simplify the deployment procedure and to make the backend portable across host servers.

### Server Security and Privacy

The app handles a large amount of sensitive data, which must be stored and transferred securely. User data were handled according to good clinical practice standards, with users being given unique anonymous identifier numbers when the app is first launched on a new device. For secure data transmission, we require the use of HTTPS across all our end points, which makes tampering with requests and responses significantly more difficult. To authorize clients, we implemented a time-based token system where the server and client have a shared secret, which is then combined with a timestamp and hashed, and then sent over the network to authenticate the user.

### Device Testing

The app was tested on multiple devices for a range of parameters to establish its suitability for and performance on a diverse range of hardware. The iOS app was tested on iPhone 6S (iOS 13.6), iPhone 6 Plus (iOS 13.2), iPhone 11 (iOS 13.7), iPhone 12 (iOS 14.6), and iPhone 13 (iOS 14.8). The Android version was tested on a range of devices including Google Pixel 4a (Android 13), Samsung Galaxy A32 (Android 12), Realme 5 Pro (Android 11), Huawei P20 Pro (Android 10), and Redmi 7 (Android 10). The app worked on all devices smoothly. Based on qualitative human experience, the app operations on the older phone models were as good as on the newer models, highlighting the low degree by which the app taxed the system's resources.

Performance testing was done using Xcode (for iOS) and Android Studio Profile (for Android) to ensure that the app does not disrupt the normal performance of the phone. During normal use, we observed that memory consumption was constant with no memory leaks, and CPU usage remained low, with the CPU only being used during tab switches and submissions.

We tested the app for battery usage on an iPhone 6 Plus and Redmi 7 for 2 months as development was carried out and for 2 weeks following installation of the finalized version of the app bundle. We deliberately chose older model phones, as they have less battery capacity, less energy-optimized hardware, and smaller batteries than more recent models. During normal use of the app, the battery consumption was mostly in the low to medium range.

### User-Centered Design Approach

Throughout the design and development process, we used a user-centered design approach [31] to ensure a user-focused, cocreation-driven app and followed a 3-step process:

1. User research: To understand the unique mental health needs, preferences, and experiences of our target users, we conducted small focus groups even during the design stage of the app. These discussions provided valuable requirements for various aspects of app design, such as sensor access, privacy concerns, user experience, and push notification preferences. By capturing this information early in the design process, we were able to ensure that our app would be both relevant and engaging for our target audience.
2. Design and prototyping: Drawing from the user research findings, our team developed design concepts for Mindcraft's visual design, UI, and key features. We created a series of low-fidelity prototypes to test and refine the app's design and functionality, ensuring that our design choices were aligned with user preferences and needs. This iterative process allowed us to optimize the app's design, making it more user-friendly and intuitive for CYP.
3. Iterative testing: To further refine Mindcraft, we held multiple user testing sessions with focus group participants. These sessions involved real-time feedback, which we used to make informed decisions about changes to the app's design and functionality. Notable decisions made during this process included reworking the UI, limiting notifications to once a day, and prioritizing the development of the progress tab.

By involving users early in the design process and maintaining open communication throughout, the user-centered design approach resulted in an app that is not only effective and engaging but also addresses the needs of our target audience.

### Ethics Approval

Ethics approval for the study was granted by the Imperial College London Research Ethics Committee (ICREC 20IC6132).

### Informed Consent

Informed consent was obtained at the start of the study, with parents providing consent for those aged younger than 16 years. Only once the participant understood the privacy policy and accepted the terms and conditions of the study in the app were they able to proceed through the app and partake in the study. The privacy policy and information storage practices were also accessible within the app.

### Privacy and Confidentiality

We ensured European Union General Data Protection Regulation compliance with data retrieval, anonymization, and a withdrawal process. All participants were informed, in the terms and conditions, of where and how the data they provided would be used. Users had full control over the data that were collected and could opt out of any single type of data collection at any time. Users had the option to delete the entirety of their data and withdraw from the study via the settings option in the app.

### Compensation

Participation was incentivized with a prize draw for a £30 (US $37) voucher and an educational session.

### Study Procedure

For user acceptance testing, participants were recruited via social media platforms (Twitter and Nextdoor, a local neighborhood site). The inclusion criteria included being aged 14-18 years, fluency in English, access to a video and audio-enabled device, and possession of a smartphone. The exclusion criterion was insufficient language skills. Participants were instructed to use the app for 3 days, explore its various features, and share their experience of using the app with the researchers following the evaluation period.

For the pilot study, we reached out to many secondary educational establishments via email and chose the first school that expressed interest in participating. Participants were recruited via this secondary school, which promoted the study directly to students through emails sent home. Inclusion and exclusion criteria for pilot testing were identical to those for user acceptance testing. The school forwarded information on the app to year 10, 11, 12, and 13 pupils.

Interested pupils voluntarily completed a web-based survey via a link provided in the promotional information. Informed consent was obtained digitally, with parents providing consent for those aged younger than 16 years. Upon completion of the survey, participants received a link to download the app from the App Store or Play Store, along with a unique login for the app. Participants were asked to use the app for 2 weeks. Upon completion of the 2-week testing period, user engagement data and the types of active and passive data shared by the users were analyzed to evaluate the app's performance.

## Results

### User Acceptance Testing

After the app's development, user acceptance testing was conducted with a recruited patient and public involvement group. We carried out an exploratory qualitative study with this group to understand the target audience's user experience and identify barriers to adopting Mindcraft. Eight young people (including 4 female individuals) aged 15-17 years, with a mean age of 15.9 (SD 0.9) years were invited to download the app and use it for a 3-day evaluation period. Two researchers conducted 2 digital patient and public involvement groups via Microsoft Teams (Microsoft), each comprising 4 participants, lasting 180 minutes. The semistructured interview explored their experience using the app.

The qualitative feedback was predominantly positive. Discussions with study participants revealed that some viewed the app as a friendly tool promoting self-awareness and personal growth. They found the app enabled them to increase their emotional awareness and gain a better understanding of themselves, as reported in previous studies on self-monitoring tools [32,33]. Participants perceived the app interface as engaging and less clinical. Although they expressed concerns regarding data privacy and sharing (particularly passive data) and desired greater control, they were relatively more comfortable sharing passive data for mental health. Our test users did not consider social competition a motivator in mental health apps, which contrasts with the findings of other studies [34,35].

Based on user feedback, we revised the app's text prompts and updated the UI to offer more control over passive sensor data collection. The updated app was uploaded to the App Store and Play Store for the pilot study with a larger user group.

### Pilot Study

The app was advertised to students from a secondary school across 4-year groups (aged 14-18 years) for a 2-week trial period. A total of 39 students (20 male students and 19 female students), with a mean age of 15.74 (SD 1.09) years, volunteered and downloaded the app. Of these, 32 used the app on iPhones, while the rest used Android phones.

**Figure 4:**
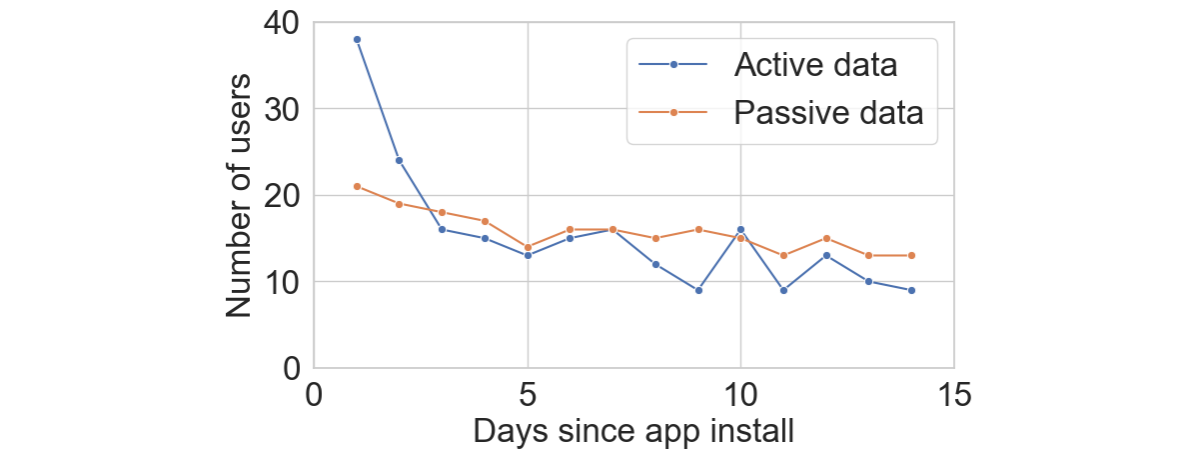
User engagement plot showing the number of users using the app (sharing active and passive data) over the 2-week period.

We calculated user engagement over time by analyzing the number of users who used the app over the 2-week evaluation period (Figure 4). Out of the 39 users, 16 (41%) used the app after 7 days, while 9 (23%) used it after 14 days. The mean number of days the app was used during the first 14 days was 5.76 (SD 4.48). Passive data collection allowed the app to gather more well-being metrics over time without user intervention. While the number of users sharing active data decreased after the first week probably because of the app's novelty wearing off, the number of users sharing passive data remained steady in the second week (Figure 4), indicating that passive data collection may offer a more sustainable data collection method in the long term.

**Figure 5:**
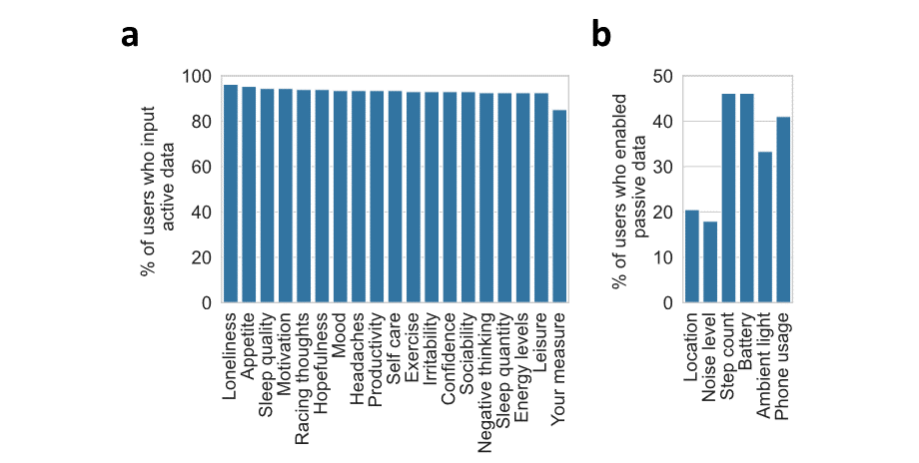
Data sharing preferences of the users. (A) Bar chart of the percentage of users sharing different types of active data. (B) Bar chart of the percentage of users sharing different types of passive data.

Seven active data questions were mandatory, while the other 12 were optional (enabled by default). Although users had the option to disable optional active data questions, 92.5% (36/39) left them enabled. Over 90% of users (36/39, 92.5%) answered all active data questions (except for the customizable Your Measure) on the days they used the app (Figure 5A). Loneliness, appetite, and sleep quality were the most frequently answered active data questions. Concerning passive trackers, users favored sharing noninvasive data, such as step count and battery levels (Figure 5B). However, they were reluctant to share intrusive passive data, like location and background noise levels, because of privacy concerns.

## Discussion

### Principal Findings

The development and initial testing of the Mindcraft app have shown promising results in monitoring mental health. The pilot study found good user retention and engagement, with 23.07% of users (9/39) using the app after 14 days. While Mindcraft's effectiveness and acceptability among the target population to track their mental health may be attributed to its user-centered design, the emphasis on transparency and privacy, and the customizable use of both active and passive data collection strategies, we recognize that our study's good retention rates should be interpreted with caution. The rates may be influenced by participants' awareness of being part of a research study, potentially leading to increased engagement compared to the naturalistic app user retention rate of 4% over a 15-day period [36,37].

Our results support previous findings [38-40], indicating that passive data collection can provide a sustainable and effective way to monitor mental health symptoms over time. We found that passive data collection reduced user burden while also enabling the collection of a wider range of well-being metrics. These benefits have important implications for the design of mental health monitoring apps, as app developers can leverage the advantages of passive data collection. By prioritizing user privacy, transparency, and customizability of data sharing, developers can design apps that foster user trust and engagement.

### Comparison With Prior Work

We acknowledge that numerous studies and apps [11,14,19,41-55] use passive sensing to support mental health and well-being. However, the majority of these studies target adult populations, while research on CYP remains limited [19,20]. Our study aims to address this gap by focusing on the mental well-being of the general adolescent population using a combined active-passive data approach. A few studies [19,43,54,55] have investigated passive sensing for adolescent mental health. Maharjan et al [55] evaluated passive sensing for predicting maternal depression in young mothers in a low-resource setting. Cao et al [43] and Mullick et al [54] examined the effectiveness of passive sensing in tracking depression symptoms in clinically depressed adolescents. The study by MacLeod et al [19] is the closest to our study, as it involved adolescents from both clinical and nonclinical settings. However, it exclusively used passive sensing, and the participants had no interactions with the study app after the initial setup. In comparison, our study focuses on monitoring the mental well-being of the general adolescent population by integrating both active and passive data. Our app enables users to decide which active and passive data they want to share, providing them with control over their privacy, a key factor for real-world deployment to the general population. We demonstrate that combining both active and passive data collection strategies, with an emphasis on privacy, transparency, and an engaging design, effectively promotes sustained engagement with the app and improves long-term data collection from the general adolescent population. Additionally, we incorporated a user-centered design approach, gathering end user feedback during the development process, an element often neglected in mental health app studies [56,57]. It is worth mentioning that, given the lack of regulatory oversight in the mental health app market [10,58], Mindcraft's development was guided by a team of experienced clinicians, ensuring evidence-based and clinically relevant content. In summary, although Mindcraft shares some similarities with other mental health apps and studies, its focus on the general adolescent population through an integrated active-passive data approach, user-centered design, and clinically relevant content highlights its potential to contribute meaningfully to the field.

### Limitations

Our study has some limitations that suggest directions for future research. Although positive results about the user engagement were observed, the conclusions must be considered preliminary because of the relatively small sample size, and larger samples are needed to validate the acceptability of the platform among the target population. Additionally, the pilot study users used the app for a limited period of 2 weeks, making it necessary to investigate its usage over a longer period to assess its effectiveness. Another limitation to consider is the potential impact of the research study environment on our retention and engagement rates. The heightened engagement we observed might be partly attributed to the participants' awareness of their involvement in a study. Therefore, future studies should aim to assess Mindcraft's retention rates in real-world settings outside the research context, to provide a more accurate reflection of its performance.

### Future Research

Throughout the study, we recognized opportunities to improve Mindcraft's user experience and capability to build a more comprehensive user behavior profile in its future version. Potential future enhancements could include gamification, which has been shown to increase user engagement in therapeutic apps [59] and mental health interventions for adolescents [60]. The app may also benefit from new passive data sources like voice recordings for speech emotion recognition and continuous accelerometer and gyroscope data for more meaningful activity measures and sleep monitoring. Integrating music listening patterns, as shown by Rickard et al [11], could further help Mindcraft better track users' emotional well-being over longer periods without being too intrusive.

Although the potential of recommended systems has been occasionally explored in health care research [61-63], the possibility of taking advantage of this technology to improve mental health care is yet to be sufficiently explored. Mindcraft presents the opportunity for an enhanced recommendation system to be developed where recommendations may be delivered based on personality, along with other factors such as demographics, behavior, and self-reported scores for specific symptoms. Future versions of Mindcraft could integrate a reinforcement learning–based personalized recommendation system, where an agent may be trained to work toward recommending activities [64,65] or mental imagery [66] to increase mood and mental health scores while being penalized and rewarded accordingly based on score variations and user feedback.

While platforms such as Kooth and Childline provide comprehensive coverage for those seeking help, these services require CYP to be motivated to engage with a counselor. Mindcraft can help overcome this motivation barrier by using passive tracking data to detect early indicators of poor well-being and mental health issues, triggering them to seek counseling help. Alternatively, the Mindcraft app could also be extended to serve as an intervention system by incorporating in-app counseling services based on the active and passive data collected. This would be a step toward achieving the goal of self-service mental health services and improving access to care for those who may not otherwise seek help.

### Conclusions

The Mindcraft app has shown promising results in monitoring mental health and promoting user engagement among CYP during its development and initial testing. The app's user-centered design, the focus on privacy and transparency, and a combination of active and passive data collection strategies have contributed to its effectiveness and acceptability among the target population. Future enhancements could include gamification, additional sensor data, and personalized interventions to improve user engagement and mental health outcomes. By continuing to refine and expand the app, the Mindcraft platform has the potential to contribute meaningfully to the field of mental health care for young people.

## Abbreviations

CYP: children and young people
UI: user interface

## Data Availability

The study data are not publicly available because of the General Data Protection Regulation restrictions and privacy policies outlined in the participant recruitment. The data sets generated during this study are available from the corresponding author upon reasonable request.

## References

[1] Westberg KH, Nyholm M, Nygren JM, Svedberg P (2022). Mental health problems among young people: a scoping review of help-seeking. Int J Environ Res Public Health.

[2] (2021). Mental health of children and young people in England. NHS Digital.

[3] Golberstein E, Wen H, Miller BF (2020). Coronavirus disease 2019 (COVID-19) and mental health for children and adolescents. JAMA Pediatr.

[4] Petrovich SB, Gewirtz JL (1985). The attachment learning process and its relation to cultural and biological evolution: proximate and ultimate considerations. The Psychobiology of Attachment and Separation.

[5] Burke AR, McCormick CM, Pellis SM, Lukkes JL (2017). Impact of adolescent social experiences on behavior and neural circuits implicated in mental illnesses. Neurosci Biobehav Rev.

[6] Teicher MH, Andersen SL, Polcari A, Anderson CM, Navalta CP (2002). Developmental neurobiology of childhood stress and trauma. Psychiatr Clin North Am.

[7] Novick AM, Levandowski ML, Laumann LE, Philip NS, Price LH, Tyrka AR (2018). The effects of early life stress on reward processing. J Psychiatr Res.

[8] Galea S, Merchant RM, Lurie N (2020). The mental health consequences of COVID-19 and physical distancing: the need for prevention and early intervention. JAMA Intern Med.

[9] Rideout V, Peebles A, Mann S, Robb MB (2021). The CommonSense census: media use by tweens and teens. CommonSenseMedia.

[10] Punukollu M, Marques M (2019). Use of mobile apps and technologies in child and adolescent mental health: a systematic review. Evid Based Ment Health.

[11] Rickard N, Arjmand HA, Bakker D, Seabrook E (2016). Development of a mobile phone app to support self-monitoring of emotional well-being: a mental health digital innovation. JMIR Ment Health.

[12] Donker T, Petrie K, Proudfoot J, Clarke J, Birch MR, Christensen H (2013). Smartphones for smarter delivery of mental health programs: a systematic review. J Med Internet Res.

[13] Gloster AT, Richard DCS, Himle J, Koch E, Anson H, Lokers L, Thornton J (2008). Accuracy of retrospective memory and covariation estimation in patients with obsessive-compulsive disorder. Behav Res Ther.

[14] Ben-Zeev D, Scherer EA, Wang R, Xie H, Campbell AT (2015). Next-generation psychiatric assessment: using smartphone sensors to monitor behavior and mental health. Psychiatr Rehabil J.

[15] Canzian L, Musolesi M (2015). Trajectories of depression: unobtrusive monitoring of depressive states by means of smartphone mobility traces analysis. https://dl.acm.org/doi/abs/10.1145/2750858.2805845.

[16] Khwaja M, Pieritz S, Faisal AA, Matic A (2021). Personality and engagement with digital mental health interventions. https://dl.acm.org/doi/abs/10.1145/3450613.3456823.

[17] Chittaranjan G, Blom J, Gatica-Perez D (2011). Who's who with big-five: analyzing and classifying personality traits with smartphones. https://ieeexplore.ieee.org/abstract/document/5959587.

[18] Khwaja M, Vaid SS, Zannone S, Harari GM, Faisal AA, Matic A (2019). Modeling personality vs. modeling personalidad. Proc ACM Interact Mob Wearable Ubiquitous Technol.

[19] MacLeod L, Suruliraj B, Gall D, Bessenyei K, Hamm S, Romkey I, Bagnell A, Mattheisen M, Muthukumaraswamy V, Orji R, Meier S (2021). A mobile sensing app to monitor youth mental health: observational pilot study. JMIR Mhealth Uhealth.

[20] Melbye S, Kessing LV, Bardram JE, Faurholt-Jepsen M (2020). Smartphone-based self-monitoring, treatment, and automatically generated data in children, adolescents, and young adults with psychiatric disorders: systematic review. JMIR Ment Health.

[21] Livingstone S, Stoilova M, Nandagiri R (2019). Children's data and privacy online: growing up in a digital age: an evidence review. LSE Research Online.

[22] ICH Harmonised Guideline (2001). Guideline for good clinical practice. J Postgrad Med.

[23] (2021). ioS - Apple. Apple Inc.

[24] Android - Open Handset Alliance. Google Inc.

[25] Chandrashekar P (2018). Do mental health mobile apps work: evidence and recommendations for designing high-efficacy mental health mobile apps. Mhealth.

[26] Lau N, O'Daffer A, Yi-Frazier JP, Rosenberg AR (2021). Popular evidence-based commercial mental health apps: analysis of engagement, functionality, aesthetics, and information quality. JMIR Mhealth Uhealth.

[27] Mental health innovations. Shout.

[28] YoungMinds. YoungMinds.

[29] Best For You. NHS.

[30] Kooth PLC. Kooth.

[31] Norman DA, Draper SW (1986). User Centered System Design: New Perspectives on Human-Computer Interaction.

[32] Kauer SD, Reid SC, Crooke AHD, Khor A, Hearps SJC, Jorm AF, Sanci L, Patton G (2012). Self-monitoring using mobile phones in the early stages of adolescent depression: randomized controlled trial. J Med Internet Res.

[33] Kenny R, Dooley B, Fitzgerald A (2015). Feasibility of "CopeSmart": a telemental health app for adolescents. JMIR Ment Health.

[34] Peng W, Kanthawala S, Yuan S, Hussain SA (2016). A qualitative study of user perceptions of mobile health apps. BMC Public Health.

[35] Anderson K, Burford O, Emmerton L (2016). Mobile health apps to facilitate self-care: a qualitative study of user experiences. PLoS One.

[36] Baumel A, Muench F, Edan S, Kane JM (2019). Objective user engagement with mental health apps: systematic search and panel-based usage analysis. J Med Internet Res.

[37] Kaveladze BT, Wasil AR, Bunyi JB, Ramirez V, Schueller SM (2022). User experience, engagement, and popularity in mental health apps: secondary analysis of app analytics and expert app reviews. JMIR Hum Factors.

[38] Torous J, Staples P, Onnela JP (2015). Realizing the potential of mobile mental health: new methods for new data in psychiatry. Curr Psychiatry Rep.

[39] Rabbi M, Ali S, Choudhury T, Berke E (2011). Passive and in-situ assessment of mental and physical well-being using mobile sensors. https://dl.acm.org/doi/abs/10.1145/2030112.2030164.

[40] Insel TR (2017). Digital phenotyping: technology for a new science of behavior. JAMA.

[41] Wahle F, Kowatsch T, Fleisch E, Rufer M, Weidt S (2016). Mobile sensing and support for people with depression: a pilot trial in the wild. JMIR Mhealth Uhealth.

[42] DeMasi O, Feygin S, Dembo A, Aguilera A, Recht B (2017). Well-being tracking via smartphone-measured activity and sleep: cohort study. JMIR Mhealth Uhealth.

[43] Cao J, Truong AL, Banu S, Shah AA, Sabharwal A, Moukaddam N (2020). Tracking and predicting depressive symptoms of adolescents using smartphone-based self-reports, parental evaluations, and passive phone sensor data: development and usability study. JMIR Ment Health.

[44] Matthews M, Abdullah S, Murnane E, Voida S, Choudhury T, Gay G, Frank E (2016). Development and evaluation of a smartphone-based measure of social rhythms for bipolar disorder. Assessment.

[45] Faurholt-Jepsen M, Vinberg M, Frost M, Christensen EM, Bardram J, Kessing LV (2014). Daily electronic monitoring of subjective and objective measures of illness activity in bipolar disorder using smartphones– the MONARCA II trial protocol: a randomized controlled single-blind parallel-group trial. BMC Psychiatry.

[46] Berrouiguet S, Ramírez D, Barrigón ML, Moreno-Muñoz P, Carmona Camacho R, Baca-García E, Artés-Rodríguez A (2018). Combining continuous smartphone native sensors data capture and unsupervised data mining techniques for behavioral changes detection: a case series of the evidence-based behavior (eb2) study. JMIR Mhealth Uhealth.

[47] Wang R, Aung MS, Abdullah S, Brian R, Campbell AT, Choudhury T, Hauser M, Kane J, Merrill M, Scherer EA, Tseng VWS, Ben-Zeev D (2016). CrossCheck: toward passive sensing and detection of mental health changes in people with schizophrenia. https://dl.acm.org/doi/abs/10.1145/2971648.2971740.

[48] Beiwinkel T, Kindermann S, Maier A, Kerl C, Moock J, Barbian G, Rössler W (2016). Using smartphones to monitor bipolar disorder symptoms: a pilot study. JMIR Mental Health.

[49] Jacobson NC, Chung YJ (2020). Passive sensing of prediction of moment-to-moment depressed mood among undergraduates with clinical levels of depression sample using smartphones. Sensors (Basel).

[50] Schueller SM, Begale M, Penedo FJ, Mohr DC (2014). Purple: a modular system for developing and deploying behavioral intervention technologies. J Med Internet Res.

[51] Gideon J, Provost EM, McInnis M (2016). Mood state prediction from speech of varying acoustic quality for individuals with bipolar disorder. https://ieeexplore.ieee.org/document/7472099.

[52] Opoku AK, Visuri A, Ferreira DS (2019). Towards early detection of depression through smartphone sensing.

[53] Dargél AA, Mosconi E, Masson M, Plaze M, Taieb F, Von Platen C, Buivan TP, Pouleriguen G, Sanchez M, Fournier S, Lledo PM, Henry C (2020). Toi Même, a mobile health platform for measuring bipolar illness activity: protocol for a feasibility study. JMIR Res Protoc.

[54] Mullick T, Radovic A, Shaaban S, Doryab A (2022). Predicting depression in adolescents using mobile and wearable sensors: multimodal machine learning-based exploratory study. JMIR Form Res.

[55] Maharjan SM, Poudyal A, van Heerden A, Byanjankar P, Thapa A, Islam C, Kohrt BA, Hagaman A (2021). Passive sensing on mobile devices to improve mental health services with adolescent and young mothers in low-resource settings: the role of families in feasibility and acceptability. BMC Med Inform Decis Mak.

[56] Aryana B, Brewster L, Nocera JA (2018). Design for mobile mental health: an exploratory review. Health Technol.

[57] Vial S, Boudhraâ S, Dumont M (2022). Human-centered design approaches in digital mental health interventions: exploratory mapping review. JMIR Ment Health.

[58] Grist R, Porter J, Stallard P (2017). Mental health mobile apps for preadolescents and adolescents: a systematic review. J Med Internet Res.

[59] Sardi L, Idri A, Fernández-Alemán JL (2017). A systematic review of gamification in e-Health. J Biomed Inform.

[60] Merry SN, Stasiak K, Shepherd M, Frampton C, Fleming T, Lucassen MFG (2012). The effectiveness of SPARX, a computerised self help intervention for adolescents seeking help for depression: randomised controlled non-inferiority trial. BMJ.

[61] Duan L, Street WN, Xu E (2011). Healthcare information systems: data mining methods in the creation of a clinical recommender system. Enterp Inf Syst.

[62] Rabbi M, Aung MH, Zhang M, Choudhury T (2015). MyBehavior: automatic personalized health feedback from user behaviors and preferences using smartphones. https://dl.acm.org/doi/10.1145/2750858.2805840.

[63] Khwaja M, Ferrer M, Iglesias JO, Faisal AA, Matic A (2019). Aligning daily activities with personality: towards a recommender system for improving wellbeing. https://dl.acm.org/doi/10.1145/3298689.3347020.

[64] Mulani J, Heda S, Tumdi K, Patel J, Chhinkaniwala H, Patel J (2020). Deep reinforcement learning based personalized health recommendations. Deep Learning Techniques for Biomedical and Health Informatics.

[65] Pieritz S, Khwaja M, Faisal AA, Matic A (2021). Personalised recommendations in mental health apps: the impact of autonomy and data sharing. https://dl.acm.org/doi/abs/10.1145/3411764.3445523.

[66] Pearson J, Naselaris T, Holmes EA, Kosslyn SM (2015). Mental imagery: functional mechanisms and clinical applications. Trends Cogn Sci.

